# Stick a Needle in My Eye: A Case Report of Penetrating Needlefish Injury Causing Cavernous Sinus Thrombosis and Carotid Cavernous Fistula

**DOI:** 10.7759/cureus.34453

**Published:** 2023-01-31

**Authors:** Maimonah Alfaheed, Sarah Campos, Joel Gupta, Jabeen Fayyaz, Sasha Litwin

**Affiliations:** 1 Emergency Medicine, The Hospital for Sick Children, Toronto, CAN; 2 Faculty of Medicine, University of Toronto, Toronto, CAN

**Keywords:** orbital cellulitis, orbital injury, pediatric emergency department (ped), cerebral vein thrombosis (cvt), traumatic carotid cavernous fistula, needlefish, case report

## Abstract

While swimming in the ocean on vacation in Cuba, a previously healthy 17-year-old female was unexpectedly stabbed through her orbit and into her brain by a needlefish. This is a unique case of a penetrating injury causing orbital cellulitis, retro-orbital abscess, cerebral venous sinus thrombosis and carotid cavernous fistula. After initial management at a local emergency department, she was transferred to a tertiary care trauma centre where she was treated by a team of emergency, neurosurgery, stroke neurology, ophthalmology, neuroradiology and infectious disease physicians. The patient faced a significant risk of a thrombotic event. There was careful consideration from the multidisciplinary team about the utility of thrombolysis or an interventional neuroradiology procedure. Ultimately, the patient was treated conservatively with intravenous antibiotics, low molecular weight heparin and observation. The patient continued to show clinical improvement several months later, which supported the challenging decision to opt for conservative management. There are very few cases to guide the treatment of this type of contaminated penetrating orbital and brain injury.

## Introduction

Cerebral venous sinus thrombosis (CVST) is a potentially life-threatening thrombus that obstructs intracranial venous drainage leading to parenchymal edema, hemorrhage and decreased perfusion [1,2]. It is a rare condition that may be encountered by emergency physicians, general practitioners, oncologists, hematologists, obstetricians, neurologists and neurosurgeons. There are many predisposing causes for CVST, including genetic or acquired pro-thrombotic conditions, use of estrogenic hormones, peripartum or post-operative states, malignancy, traumatic injury and infection, including orbital cellulitis [1,2]. A carotid cavernous fistula (CCF) is an abnormal connection between the cavernous sinus and the carotid arterial system. CCF is a very rare complication of head trauma, especially basal skull fracture [3,4]. We report a unique case of a penetrating brain injury by a needlefish causing orbital cellulitis, retro-orbital abscess, CVST and CCF. CVST and CCF can lead to stroke, visual impairment and mortality if not recognized and treated. There are few previous case reports of penetrating orbital and brain injury caused by needlefish [5-9].

## Case presentation

**Figure 1:**
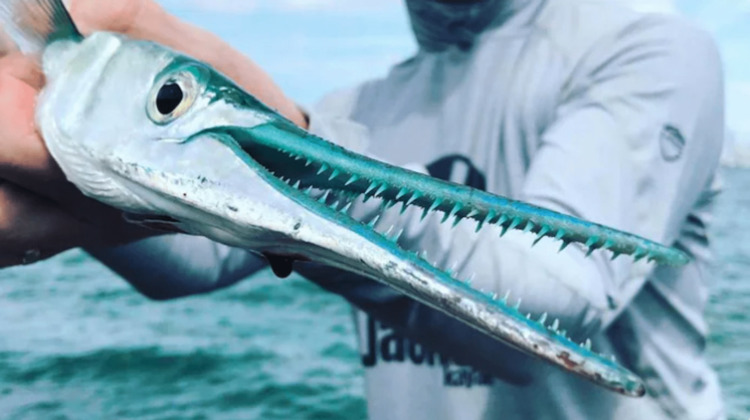
Needlefish. Photo credit: Mark Radcliffe

A previously healthy 17-year-old female was swimming in the ocean while on vacation in Cuba when a needlefish jumped out of the water and penetrated her right lateral eyelid with its long, narrow jaws. The needlefish remained impaled in her eyelid. The patient swam to the shore and immediately pulled the fish out of her eyelid. She did not have any initial impairment in her vision or an associated penetrating globe injury. Residual teeth and foreign materials from the fish remained in the wound (Figure 1).

**Figure 2:**
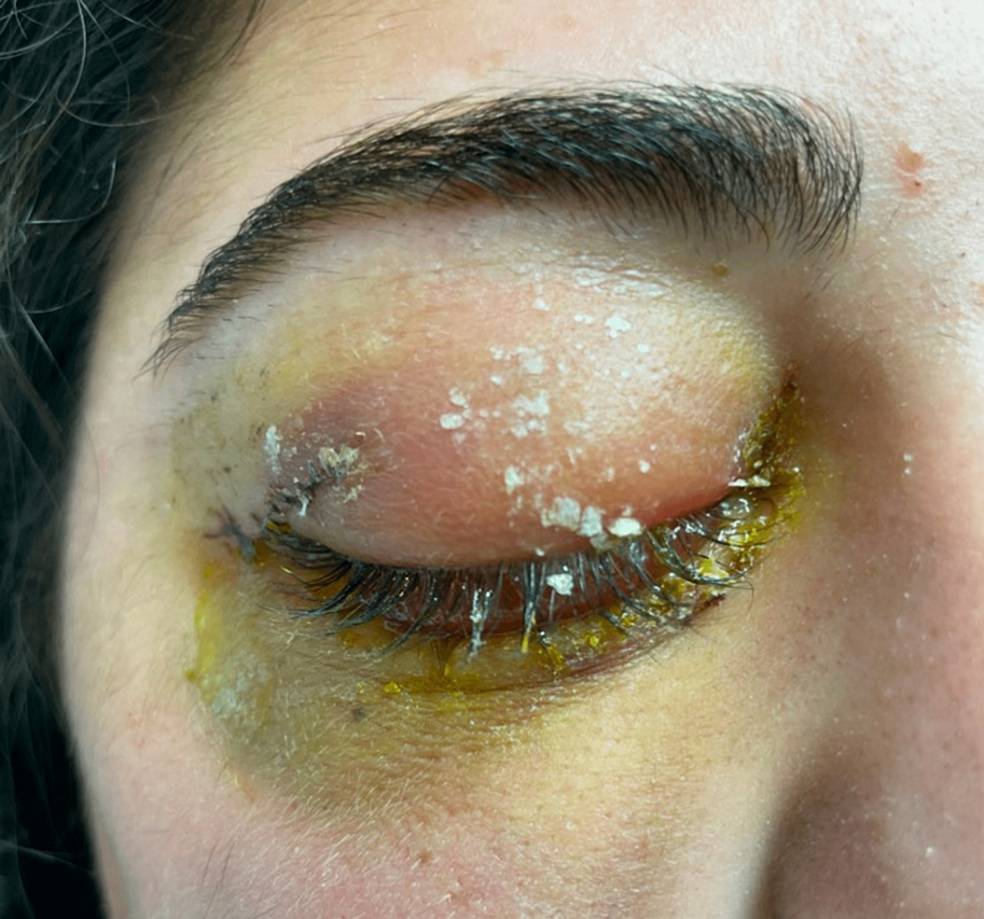
Right periorbital edema, discharge and ecchymosis.

Later that day, she attended a local emergency department where the superficial laceration was sutured. No systemic or topical antibiotics were started at that time. When she returned home to Canada two days later, she started to develop eye pain, periorbital swelling and purulent discharge from the laceration site. She presented to a local emergency department with significant periorbital pain and swelling (Figure 2).

A computed tomography (CT) scan of her orbits done at her local emergency department showed orbital cellulitis and a retro-orbital abscess from a penetrating injury by the long, sharp jaw of the needlefish. There was no evidence of globe injury clinically or on imaging. Routine laboratory investigations were unremarkable (complete blood count, coagulation profile). She was started on broad-spectrum intravenous antibiotics (ceftriaxone, meropenem, doxycycline and vancomycin) and was transferred to a tertiary care trauma centre. On arrival at the trauma centre, she was alert and oriented. Her vital signs were stable and within normal limits for her age. Examination of her right eye revealed significant periorbital swelling, conjunctival chemosis and a 1 cm laceration of her superior eyelid repaired with three simple interrupted sutures. The laceration was draining purulent discharge. There was no sign of globe injury. Her neurological examination revealed complete ophthalmoplegia, cranial nerve polyneuropathy with proptosis and dilated non-reactive right pupil, and loss of sensation in V1 distribution (CN3, 4, 6 and V1). Her visual acuity was significantly impaired on the right (20/200).

**Figure 3:**
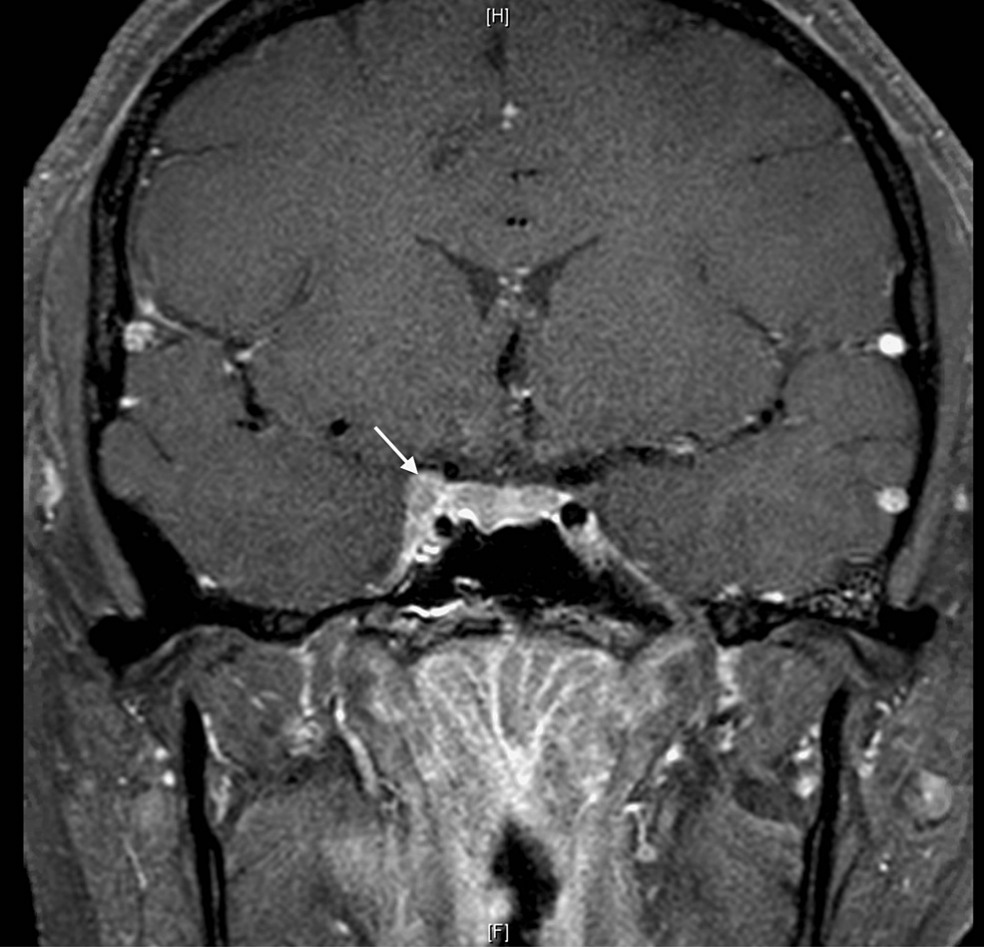
MRI of CSVT: The post-GAD T1 shows a possible filling defect within the right cavernous sinus (white arrow). MRI: magnetic resonance imaging CVST: cerebral venous sinus thrombosis GAD: Gadolinium contrast

**Figure 4:**
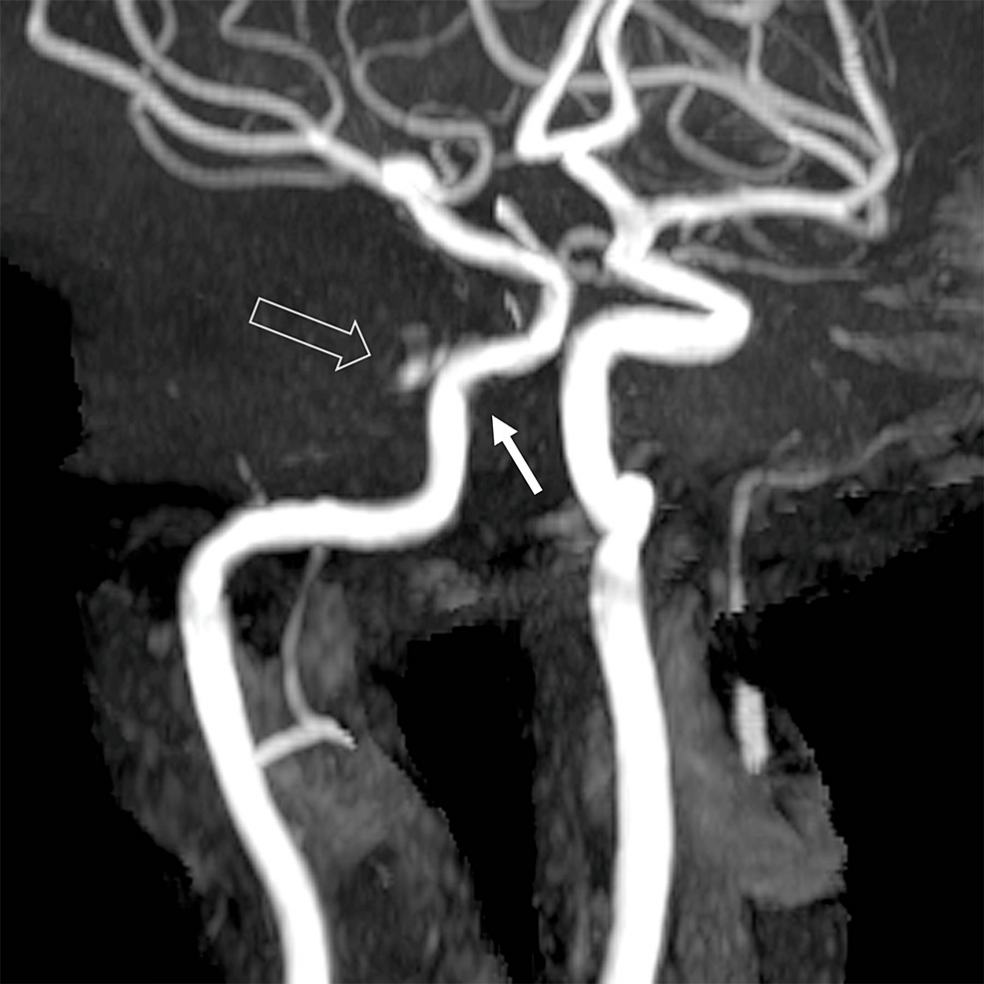
MRA of CCF: Oblique 3D time-of-flight MRA demonstrates narrowing of the right ICA (white arrow). Focal outpouching extending from ICA (open arrow) reflects the CCF. MRA: magnetic resonance angiography CCF: caroticocavernous fistula ICA: internal carotid artery

Based on the CT results from the local hospital, the neuroradiology team recommended further neuroimaging. The patient had an MRI brain with contrast and phase contrast MR venography, time-of-flight MR angiography (MRA) and CT angiography. Her initial MRI brain revealed pre- and post-septal orbital cellulitis with focal infarction, hyperdense fragments along the lateral orbit which were suspected to be foreign bodies, a linear non-displaced fracture of the posterolateral wall of her right orbit and a filling defect along the anterior and mid aspect of the right cavernous sinus representing a right CSVT (Figure 3). The MRA showed a narrowing of the right internal carotid artery (ICA) and focal outpouching from ICA reflecting the CCF (Figure 4). A subsequent conventional cerebral angiogram confirmed the presence of the CCF from the meningohypophyseal trunk of the right cavernous ICA to the posterior chamber of the right cavernous sinus.

Broad-spectrum intravenous antibiotics to cover typical microbes, as well as aquatic microbes including *Vibrio* and *Aeromonas* species (vancomycin and meropenem), and empiric antifungal medications (amphotericin) were initiated on the recommendation of the infectious diseases team. The patient was additionally given a tetanus booster and tetanus immunoglobulin. The ophthalmology team explored the wound by blunt dissection under local anesthetic to remove the superficial foreign bodies (the needlefish teeth) from the right lateral canthus and surrounding periorbital area.

There were several issues in the acute management of her injury, including the potential need for thrombolysis and antimicrobial selection. Both CVST and CCF put the patient at a very high risk of stroke. The neurosurgery and neurology stroke teams guided the anticoagulation plan and the decision to forego interventional procedures. The patient was initially observed in the pediatric intensive care unit for neuromonitoring and remained in the hospital for a total of five weeks. She received therapeutic anticoagulation therapy with unfractionated heparin at a dose of 13 u/kg/hr to target therapeutic anti-Xa levels of 0.35-0.75 (IU/mL) for approximately eight days. It was stopped after five days by expert consensus in order to ensure adequate hemostasis between the arterial and venous limbs of the CCF, acknowledging the provoked nature of the thrombus. She completed the above course of broad-spectrum antimicrobials for a total of six weeks.

About one month after her admission, repeat cerebral angiography showed interval resolution of the CCF, and repeat MRI with angiography showed improved orbital inflammation and interval improvement in the CVST. Over the following six months, her extra-ocular muscles and vision slowly improved, with her best visual acuity to date being 20/60. She continued to struggle with low mood as a result of her ongoing visual impairment.

## Discussion

In this rare case, a penetrating orbital injury by a needlefish caused a traumatic CVST and CCF. Penetrating trauma from needlefish is a rare occurrence in humans. There are other published case reports of penetrating needlefish injuries to the spinal cord [10], abdomen [11], brain and orbits [5-9].

CVST and CCF are rare complications of penetrating head trauma [3,4]. The diagnoses of CVST and CCF are difficult even with advanced neuroimaging because the signs and symptoms can be varied and non-specific. There is a lack of research on the presentation and optimal treatment of both CVST and CCF. When patients present with symptoms of CVST, such as headache or visual changes, the treating physician may request head imaging. Unfortunately, a normal non-contrast CT scan does not rule out CVST [1]. CT venogram (CTV) and contrast-enhanced MR venogram (MRV) are much more sensitive. MRV is the preferred diagnostic neuroimaging modality as it can demonstrate edema and venous infarction of the superficial and deep venous system of the brain parenchyma [12]. Interventional radiological methods such as direct cerebral venography may be necessary when MRV or CTV results are inconclusive, or if the patient requires an endovascular procedure [13].

In this case, the treating medical team had nuanced discussions about the risks and benefits of both anticoagulation therapy and the possibility of thrombolysis. The goal of anticoagulation therapy in CVST is to prevent expansion and facilitate recanalization. Thrombolysis is administered for thrombus dissolution but comes with a high rate of local and systemic bleeding. However, this scenario is exceedingly rare and the use of anticoagulation for CVST combined with CCF is not supported by large research studies. A small randomized controlled study comparing anticoagulant therapy with placebo in patients with CVST demonstrated a non-significant but favourable outcome for patients treated with anticoagulants [14-16]. Alternatively, treatment of CCF may be conservative or interventional (endovascular intervention or neurosurgery) depending on the case [17,18]. Finally, source control for the infection was essential. The patient was treated with high-dose broad-spectrum antimicrobial therapy and all superficial foreign bodies were removed. In other cases of septic or infection-related cavernous sinus thrombosis, broad-spectrum intravenous antibiotics are recommended, along with drainage of infectious fluid collections and removal of foreign bodies [19].

## Conclusions

This is a unique case of a penetrating brain injury by a needlefish causing orbital cellulitis, CVST and CCF. CVST and CCF are life-threatening conditions. Clinical features vary depending on the severity and location of the thrombosis or fistula. It is essential to expedite imaging (CTV, MRV and cerebral angiography) and refer the patient to a tertiary care trauma centre. Treatment decisions should be made by a multidisciplinary team to determine the best antimicrobial and anticoagulant (plus/minus thrombolytic) therapy for each patient presenting with a CVST and CCF.
